# Assessing Biofuel Crop Invasiveness: A Case Study

**DOI:** 10.1371/journal.pone.0005261

**Published:** 2009-04-22

**Authors:** Christopher Evan Buddenhagen, Charles Chimera, Patti Clifford

**Affiliations:** Pacific Cooperative Studies Unit, University of Hawaii at Manoa, Honolulu, Hawaii, United States of America; Stanford University, United States of America

## Abstract

**Background:**

There is widespread interest in biofuel crops as a solution to the world's energy needs, particularly in light of concerns over greenhouse-gas emissions. Despite reservations about their adverse environmental impacts, no attempt has been made to quantify actual, relative or potential invasiveness of terrestrial biofuel crops at an appropriate regional or international scale, and their planting continues to be largely unregulated.

**Methodology/Principal Findings:**

Using a widely accepted weed risk assessment system, we analyzed a comprehensive list of regionally suitable biofuel crops to show that seventy percent have a high risk of becoming invasive versus one-quarter of non-biofuel plant species and are two to four times more likely to establish wild populations locally or be invasive in Hawaii or in other locations with a similar climate.

**Conclusions/Significance:**

Because of climatic and ecological similarities, predictions of biofuel crop invasiveness in Hawaii are applicable to other vulnerable island and subtropical ecosystems worldwide. We demonstrate the utility of an accessible and scientifically proven risk assessment protocol that allows users to predict if introduced species will become invasive in their region of interest. Other evidence supports the contention that propagule pressure created by extensive plantings will exacerbate invasions, a scenario expected with large-scale biofuel crop cultivation. Proactive measures, such as risk assessments, should be employed to predict invasion risks, which could then be mitigated via implementation of appropriate planting policies and adoption of the “polluter-pays” principle.

## Introduction

There is growing interest in biofuels as a “green”, renewable solution to the world's energy needs, particularly in the face of increasing cost and declining availability of fossil fuels, and concerns over greenhouse-gas emissions and concomitant climate change. Biofuel crops continue to be promoted and planted worldwide despite questions concerning their adverse environmental impacts, inability to meet energy needs or emission requirements, and alleged non-profitability [1], [2], [3], [4]. Some evidence suggests that biofuel crops are selected for traits that contribute to a higher probability of naturalization and invasiveness [5], [6], [7], [8]. These studies, while valuable for drawing attention to the problem, are generally descriptive, rather than quantitative, or limited to the analysis of only a few species. Meanwhile, invasive species impacts are being manifested worldwide, incurring massive economic costs for their management and control and affecting landscape-level change and losses to biodiversity, especially on islands [9], [10], [11].

Tools exist to mitigate the impacts of intentional terrestrial plant introductions. A weed risk assessment system (WRA) for screening out potentially invasive species was developed and is being successfully applied in Australia [12]. Species are scored according to a set of 49 criteria, with those falling above or below a certain threshold designated as high or low risk, and accepted or rejected for importation [13]. Some species fall into the intermediate category of “evaluate” when evidence of risk is inconclusive [14]. Use of the WRA provides net economic benefits by allowing authorities to screen out costly invasive species, even after accounting for lost revenue from the small percentage of valuable non-weeds that may be incorrectly rejected [15]. The system has since been adapted for use around the world, and successfully identifies major invaders 90% and non-invaders 70% of the time [16]. Most risk assessment systems draw on a similar mix of criteria related to climate suitability, biology, undesirable characteristics and invasion history [16], [17]. There are no single plant characteristic that consistently and conclusively predict invasiveness in a species, so the WRA employs a “catch-all” approach to the evidence to improve accuracy of predictions [12]. We aimed to quantify actual, relative or potential invasiveness of biofuel crops at an appropriate regional and pan-tropical scale. To do this we documented naturalization and invasiveness in Hawaii and climatically similar regions elsewhere. We also determined potential risk using the WRA adapted for Hawaii and the Pacific regions (HPWRA) [14]. We compared invasion risks of a comprehensive list of 40 biofuel crops proposed for Hawaii versus a random sample of 40 introduced non-biofuel plant species. Our results conclusively demonstrate that actual and potential invasiveness differed significantly between proposed biofuel crops and introduced non-biofuel species.

## Results and Discussion

Compared to the sample of introduced non-biofuel species, biofuel crops were two to four times more likely to be naturalized or invasive in Hawaii or elsewhere (Table 1). Of the 40 biofuel species recommended for use in Hawaii, 58% were already naturalized there, while the random selection of 40 introduced non-biofuel species yielded only a 13% naturalization rate. Species-specific traits could explain this, but other factors are known to contribute to naturalization and invasiveness, including characteristics of the receiving environment, climate suitability, residence time, time to maturity, and degree of cultivation or propagule pressure [18], [19]. The pattern also holds true when considering invasiveness of these biofuel crops elsewhere in the world. The biofuel species included in this study were three times as likely to be invasive somewhere in the world as the introduced species (60 vs. 20%, Table 1). A similar pattern held (32 vs. 13%) for the species known to be invasive in Hawaii, but the difference was not significant (binomial test χ^2^ = 3.1176, df = 1, p-value = 0.077).

**Table 1:** The number (percentages) of biofuel crops (n = 40) and a random selection of introduced (n = 40) species with their invasiveness status in this study; to calculate percentages for biofuels naturalized and invasive in Hawaii we use biofuel species present in Hawaii for the denominator (i.e., 38).

Status	biofuel	random
Present in Hawaii	38 (95%)	40 (100%)
Naturalized in Hawaii	22 (58%)	5 (13%) **
Invasive in Hawaii	12 (32%)	5 (13%)
Invasive elsewhere	24 (60%)	8 (20%) **

** Binomial proportion tests significant at the 0.001 level.

**Figure 1:**
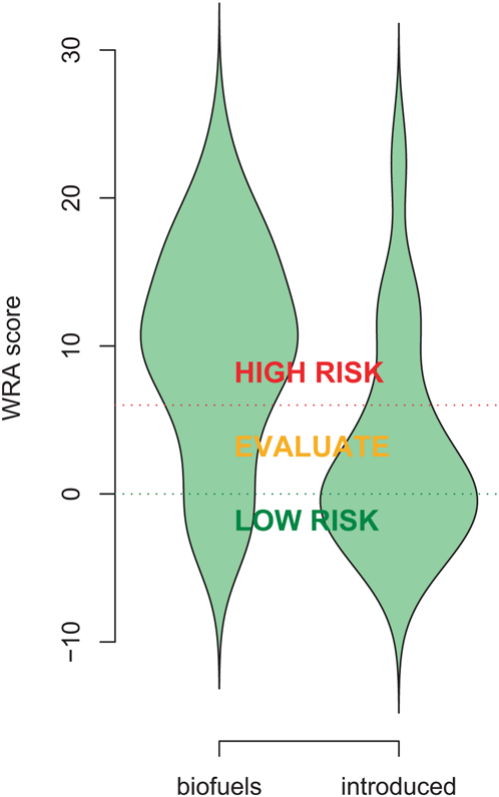
Density distributions of WRA scores of biofuels compared to a random selection of other introduced species; scores were significantly different (Wilcoxon exact test W = 1135.5 p<0.001).

Biofuel crops had HPWRA scores that skewed higher and tended to fall above the threshold for high risk species (over 6) whereas the random sample of introduced non-biofuel species was weighted toward the low-risk (below 0) end of the spectrum (Fig. 1). After the second screening, all known invaders in Hawaii and elsewhere were categorized as high risk (Tables 1, 2 and 3). Using the HPWRA, fifteen biofuel species not yet known to be invasive in Hawaii were identified as high risk compared to five of the introduced non-biofuel species. We could not find enough published information to complete two introduced species assessments (Table 2 and 3), suggesting that the WRA may not be effective at predicting invasiveness for poorly studied species.

**Table 2:** Numbers (percentages) of species falling into the WRA risk categories within a group of species proposed as biofuels and a random selection of introduced species in Hawaii.

Risk category	biofuel	random
High	28 (70%)	10 (25%)
Evaluate	3 (8%)	4 (10%)
Low	9 (22%)	24 (60%)
not assessable	0 (0%)	2 (5%)

**Table 3:** List of biofuels (n = 40) and introduced species (n = 40) in the Hawaiian Islands (HI) analyzed in this study, with associated Weed Risk Assessment (WRA) scores, naturalization (Nat) or invasive (Inv) status, biofuel use and risk category (H = High, L = Low, E = Evaluate*, NA = Not Assessable).

Species	Family	Present in HI	Nat HI	Inv HI	Inv elsewhere	Biofuel use	WRA	Risk	Ref.
*Aleurites moluccana*	Euphorbiaceae	Y	Y	Y	Y	Biodiesel	12	H	[33]
*Arachis glabrata*	Fabaceae	Y	N	N	N	Biodiesel	−1	L	[33]
*Azadirachta indica*	Meliaceae	Y	N	N	Y	Biodiesel	10	H	[33]
*Brassica napus*	Brassicaceae	N	N	N	Y	Biodiesel	16	H	[33]
*Cocos nucifera*	Arecaceae	Y	Y	N	N	Biodiesel	−4	L	[33]
*Copaifera langsdorfii*	Fabaceae	Y	N	N	N	Biodiesel	4	E	[33]
*Elaeis guineensis*	Arecaceae	Y	N	N	N	Biodiesel	9	H	[34]
*Euphorbia lathyris*	Euphorbiaceae	N	N	N	Y	Biodiesel	8	H	[33]
*Glycine max*	Fabaceae	Y	N	N	N	Biodiesel	−3	L	[33]
*Helianthus annuus*	Asteraceae	Y	Y	N	Y	Biodiesel	10.5	H	[33]
*Jatropha curcas*	Euphorbiaceae	Y	Y	N	Y	Biodiesel	17	H	[33]
*Linum usitatissimum*	Linaceae	Y	N	N	Y	Biodiesel	9.5	H	[33]
*Moringa oleifera*	Moringaceae	Y	N	N	N	Biodiesel	1	L	[33]
*Persea americana*	Lauraceae	Y	Y	N	N	Biodiesel	3	L	[33]
*Pittosporum resiniferum*	Pittosporaceae	Y	N	N	N	Biodiesel	6	E	[33]
*Pongamia pinnata*	Fabaceae	Y	N	N	Y	Biodiesel	9	H	[33]
*Ricinus communis*	Euphorbiaceae	Y	Y	Y	Y	Biodiesel	21	H	[33]
*Simmondsia chinensis*	Simmondsiaceae	Y	N	N	N	Biodiesel	−3	L	[33]
*Triadica sebifera*	Euphorbiaceae	Y	N	N	Y	Biodiesel	14	H	[33]
*Ulex europaeus*	Fabaceae	Y	Y	Y	Y	Biodiesel	20	H	[33]
*Arundo donax*	Poaceae	Y	N	N	Y	Biomass	12	H	[35]
*Calotropis gigantea*	Apocynaceae	Y	Y	N	Y	Biomass	15	H	[7]
*Cannabis sativa*	Cannabaceae	Y	Y	N	N	Biomass	11.5	H	[35]
*Casuarina equisetifolia*	Casuarinaceae.	Y	Y	Y	Y	Biomass	15	H	[34]
*Eucalyptus globulus*	Myrtaceae	Y	Y	Y	Y	Biomass	10	H	[34]
*Eucalyptus grandis*	Myrtaceae	Y	N	N	Y	Biomass	11	H	[34]
*Eucalyptus robusta*	Myrtaceae	Y	Y	N	N	Biomass	3	L	[34]
*Eucalyptus saligna*	Myrtaceae	Y	Y	N	N	Biomass	7	H	[36]
*Eucalyptus urophylla*	Myrtaceae	Y	N	N	N	Biomass	6	E	[36]
*Fraxinus uhdei*	Oleaceae	Y	Y	Y	Y	Biomass	11	H	[34]
*Macadamia integrifolia*	Proteaceae	Y	N	N	N	Biomass	−1	L	[35]
*Paraserianthes falcataria*	Fabaceae	Y	Y	Y	Y	Biomass	8	H	[34]
*Prosopis juliflora*	Fabaceae	Y	Y	Y	Y	Biomass	19	H	[37]
*Psidium cattleianum*	Myrtaceae	Y	Y	Y	Y	Biomass	18	H	[34]
*Leucaena leucocephala*	Fabaceae	Y	Y	Y	Y	Ethanol	15	H	[34]
*Panicum maximum*	Poaceae	Y	Y	Y	Y	Ethanol	17	H	[7]
*Panicum virgatum*	Poaceae	Y	Y	N	N	Ethanol	11	H	[8]
*Pennisetum purpureum*	Poaceae	Y	Y	Y	Y	Ethanol	16	H	[35]
*Pueraria montana*	Fabaceae	Y	Y	N	Y	Ethanol	24	H	[38]
*Saccharum officinarum*	Poaceae	Y	N	N	N	Ethanol	−2	L	[35]
*Allium sativum*	Alliaceae	Y	N	N	N	None	−4	L	None
*Alluaudia procera*	Didiereaceae	Y	N	N	N	None	−7	L	None
*Balaka longirostris*	Arecaceae	Y	N	N	N	None	0	L	None
*Callicarpa japonica*	Verbenaceae	Y	N	N	N	None	5	L	None
*Callistemon viminalis*	Myrtaceae	Y	N	N	N	None	3	L	None
*Cardiospermum halicacabum*	Sapindaceae	Y	Y	Y	Y	None	12	H	None
*Carpobrotus edulis*	Aizoaceae	Y	N	N	Y	None	9.5	H	None
*Cleistocactus baumannii*	Cactaceae	Y	N	N	N	None	−4	L	None
*Colpothrinax wrightii*	Arecaceae	Y	N	N	N	None	−2	L	None
*Davallia fejeensis*	Davalliaceae	Y	N	N	N	None	6	H	None
*Dictyosperma album*	Arecaceae	Y	N	N	N	None	−3	L	None
*Dolichandrone spathacea*	Bignoniaceae	Y	N	N	N	None	−5	L	None
*Episcia dianthiflora*	Gesneriaceae	Y	N	N	N	None	−2	L	None
*Erythrina sigmoidea*	Fabaceae	Y	N	N	N	None	6	E	None
*Eucalyptus yarraensis*	Myrtaceae	Y	N	N	N	None	1	L	None
*Excoecaria indica*	Euphorbiaceae	Y	N	N	N	None	1	E	None
*Gardenia augusta*	Rubiaceae	Y	N	N	N	None	0	L	None
*Godmania aesculifolia*	Bignoniaceae	Y	N	N	N	None	−3	L	None
*Haplophragma adenophyllum*	Bignoniaceae	Y	N	N	N	None	0	L	None
*Hedera algeriensis*	Araliaceae	Y	N	N	N	None	3	H	None
*Laccospadix australasica*	Arecaceae	Y	N	N	N	None	1	E	None
*Lantana camara*	Verbenaceae	Y	Y	Y	Y	None	21	H	None
*Leea guineensis*	Vitaceae	Y	N	N	N	None	−1	L	None
*Mimosa diplotricha*	Fabaceae	Y	N	N	Y	None	24	H	None
*Muntingia calabura*	Elaeocarpaceae	Y	N	N	Y	None	12	H	None
*Passiflora quadrangularis*	Passifloraceae	Y	Y	Y	Y	None	11	H	None
*Philodendron variifolium*	Araceae	Y	N	N	N	None	NA	NA	None
*Pithecellobium dulce*	Fabaceae	Y	Y	Y	Y	None	14	H	None
*Ruttya fruticosa*	Acanthaceae	Y	N	N	N	None	0	L	None
*Sabal mauritiiformis*	Arecaceae	Y	N	N	N	None	−2	L	None
*Schefflera crassifolia*	Araliaceae	Y	N	N	N	None	NA	NA	None
*Senecio mandraliscae*	Asteraceae	Y	N	N	N	None	−3	L	None
*Serianthes kanehirae*	Fabaceae	Y	N	N	N	None	−5	L	None
*Solanum capsicoides*	Solanaceae	Y	Y	Y	Y	None	15	H	None
*Stromanthe macrochlamys*	Marantaceae	Y	N	N	N	None	0	L	None
*Strophanthus amboensis*	Apocynaceae	Y	N	N	N	None	2	E	None
*Syngonium auritum*	Araceae	Y	N	N	N	None	1	L	None
*Tabebuia roseo-alba*	Bignoniaceae	Y	N	N	N	None	3	L	None
*Tabernaemontana elegans*	Apocynaceae	Y	N	N	N	None	−1	L	None
*Thymus vulgaris*	Lamiaceae	Y	N	N	N	None	6	L	None

* Risk designation for WRA scores of 1–6 follows use of a secondary screening developed by Daehler et al. [14].

Both the biofuel crops and introduced species in our analysis were presumably chosen for importation and cultivation in part because of their climatic suitability to subtropical islands (i.e., Hawaii). In any case all but two of the biofuel and all the non-biofuel species already grow in Hawaii. We contribute quantitative evidence that, compared to other plants, biofuel crops are selected for many of the same traits identified in successful invasive species, as supported by other authors [5], [6], [7], [8]. Apparently invasion probability and desirability as biofuel can relate to traits shared across plant families, and 25 (63%) of the biofuel crops are in weedy families Euphorbiaceae, Fabaceae, Myrtaceae, Poaceae [20], [21], [22]. Species traits identified by the WRA have been shown to relate to actual invasiveness [16], [23] provided they interact suitably with local environmental conditions, but propagule pressure is also known to contribute strongly to invasion success [24], [25]. Widespread planting of biofuel crops will increase propagule pressure tremendously, and in combination with an effective dispersal mechanism, increases the probability of invasion by the high-risk species identified in this study (Table 3). For the fifteen high risk species not currently naturalized in the Hawaiian Islands, invasion and associated problems could be manifested more quickly than in temperate regions, due to a propensity for greatly reduced lag-times in tropical climates [19]. Risk assessments using a standardized methodology should help decision makers to evaluate biofuel crops. Not all ‘high-risk’ species are likely to be equally problematic; certain species might be rejected outright, whereas the planting of others could be regulated with proactive, precautionary measures. Though a large proportion of high risk biofuel species have already shown their invasive potential somewhere in the world, some do so only in particular circumstances, e.g., along riparian zones, or in the presence of particular dispersers and pollinators [7]. Spread may be predictably slow, or regionally controllable. Such species could potentially be planted and their invasiveness limited through conscious management and planting plans designed to prevent or reduce spread. The benefits of planting a biofuel species could outweigh the environmental costs in some circumstances, despite the risk of invasiveness [26].

There are well-documented examples of speculative agricultural introductions not only failing to meet expectations but also leading to unintended invasions and associated problems, particularly in the case of introduced pasture and legume species [27], [28]. Considering the experimental nature of many biofuel crops and the uncertainty that they will be profitable, the utility of some species has likely been overestimated and underperforming or unpromising crops will inevitably be abandoned due to the vagaries of the market. The costs of managing the impacts of persistent or spreading species in the environment, particularly following landscape-level changes associated with large scale agricultural ventures, would then be passed onto the wider community. As in other locations, only a small proportion of known invasive species are regulated in Hawaii, and policies favor entrepreneurship; private land-owners are able to plant almost any crop they choose. To mitigate these costs, the “polluter-pays” principle is one solution that could be sensibly employed by regulatory agencies approving planting programs for high-risk species [29]. High risk species could be designated as noxious weeds which require permission to plant. Proponents of high risk species could be required to pay a bond to fund control of escaping crops, or they could be required to do the control themselves at their own expense.

## Materials and Methods

We documented all terrestrial plant species identified in the literature for potential biofuel use in Hawaii (Table 3). Species already growing in Hawaii but proposed as biofuels elsewhere were also included in our analysis. Biofuel crops were defined broadly and included plants that produce energy directly via burning methods such as gasification or indirectly through conversion to liquid fuels, e.g. bio-diesel or ethanol. Any plant material could conceivably be used to produce energy, but we focused on those species that published experts considered to be the most promising. A comparison dataset included an equal number (n = 40) of randomly selected non-biofuel plant species known to be introduced in Hawaii (in cultivation or in the wild) [30], [31]. For both biofuels and these introduced species, we documented their naturalization and invasion status in Hawaii and invasiveness in climatically similar areas elsewhere (Table 1 and 3). A widely used weed risk assessment system adapted for use in Hawaii and the Pacific (HPWRA) was used to collate weed risk assessment scores for both the introduced and biofuel species [13], [14], [16]. We compared scores and the numbers falling into the three risk assessment categories (High/Reject, Evaluate, Low/Accept), following standard HPWRA methods [14] (Table 1). Species initially falling into the evaluate category were run through a second screening procedure which improves detection of high risk species [16]. Risk outcomes for each species were recorded in Table 3. Two cultivated species could not be assessed fully as there was insufficient published information to answer the minimum number of questions required by the HPWRA; these were given their own category of “not assessable” (Table 2 and 3). Score density distributions were plotted using violin plots (Figure 1). Binomial proportion tests and Wilcoxon's exact tests were used to compare categorical data (Table 2). All statistics were carried out using R version 2.7.2 [32].
